# Supplementary material for: HIVprotI: an integrated web based platform for prediction and design of HIV proteins inhibitors
Source: J Cheminform. 2018 Mar 9;10:12. doi: 10.1186/s13321-018-0266-y (PMC5845081; doi:10.1186/s13321-018-0266-y)
Supplement: Supplementary file 2 — Additional file 2. Source code of HIVProtI web server. [file 13321_2018_266_MOESM2_ESM.zip › HIVprotI_Source-code/hivprotI/weka-3-6-6/doc/weka/filters/unsupervised/attribute/NumericTransform.html]

NumericTransform


---


|  |  |  |
| --- | --- | --- |
| **Overview** \| **Package** \| **Class** \| **Tree** \| **Deprecated** \| **Index** \| **Help** \| **Weka's home** | |  |
| **PREV CLASS**   **NEXT CLASS** | **FRAMES**    **NO FRAMES**     **All Classes** |  |
| SUMMARY: NESTED \| FIELD \| CONSTR \| METHOD | DETAIL: FIELD \| CONSTR \| METHOD |  |


---


## weka.filters.unsupervised.attribute Class NumericTransform

```
java.lang.Object
  weka.filters.Filter
      weka.filters.unsupervised.attribute.NumericTransform
```

**All Implemented Interfaces:**: java.io.Serializable, CapabilitiesHandler, OptionHandler, RevisionHandler, StreamableFilter, UnsupervisedFilter

---

``` public class NumericTransform extends Filter implements UnsupervisedFilter, StreamableFilter, OptionHandler ```

Transforms numeric attributes using a given transformation method.


Valid options are:

```
 -R <index1,index2-index4,...>
  Specify list of columns to transform. First and last are
  valid indexes (default none). Non-numeric columns are 
  skipped.
```

```
 -V
  Invert matching sense.
```

```
 -C <string>
  Sets the class containing transformation method.
  (default java.lang.Math)
```

```
 -M <string>
  Sets the method. (default abs)
```

**Version:**
:   $Revision: 5543 $

**Author:**
:   Eibe Frank (eibe@cs.waikato.ac.nz)

**See Also:**: Serialized Form

---

**Constructor Summary**

`NumericTransform()`
  
          Default constructor -- sets the default transform method
to java.lang.Math.abs().


| **Method Summary** | |
| --- | --- |
| `java.lang.String` | `attributeIndicesTipText()`             Returns the tip text for this property |
| `java.lang.String` | `classNameTipText()`             Returns the tip text for this property |
| `java.lang.String` | `getAttributeIndices()`             Get the current range selection |
| `Capabilities` | `getCapabilities()`             Returns the Capabilities of this filter. |
| `java.lang.String` | `getClassName()`             Get the class containing the transformation method. |
| `boolean` | `getInvertSelection()`             Get whether the supplied columns are to be transformed or not |
| `java.lang.String` | `getMethodName()`             Get the transformation method. |
| `java.lang.String[]` | `getOptions()`             Gets the current settings of the filter. |
| `java.lang.String` | `getRevision()`             Returns the revision string. |
| `java.lang.String` | `globalInfo()`             Returns a string describing this filter |
| `boolean` | `input(Instance instance)`             Input an instance for filtering. |
| `java.lang.String` | `invertSelectionTipText()`             Returns the tip text for this property |
| `java.util.Enumeration` | `listOptions()`             Returns an enumeration describing the available options. |
| `static void` | `main(java.lang.String[] argv)`             Main method for testing this class. |
| `java.lang.String` | `methodNameTipText()`             Returns the tip text for this property |
| `void` | `setAttributeIndices(java.lang.String rangeList)`             Set which attributes are to be transformed (or kept if invert is true). |
| `void` | `setAttributeIndicesArray(int[] attributes)`             Set which attributes are to be transformed (or kept if invert is true) |
| `void` | `setClassName(java.lang.String name)`             Sets the class containing the transformation method. |
| `boolean` | `setInputFormat(Instances instanceInfo)`             Sets the format of the input instances. |
| `void` | `setInvertSelection(boolean invert)`             Set whether selected columns should be transformed or not. |
| `void` | `setMethodName(java.lang.String name)`             Set the transformation method. |
| `void` | `setOptions(java.lang.String[] options)`             Parses a given list of options. |

**Methods inherited from class weka.filters.Filter**

`batchFilterFile, batchFinished, filterFile, getCapabilities, getOutputFormat, isFirstBatchDone, isNewBatch, isOutputFormatDefined, makeCopies, makeCopy, numPendingOutput, output, outputPeek, toString, useFilter, wekaStaticWrapper`

**Methods inherited from class java.lang.Object**

`equals, getClass, hashCode, notify, notifyAll, wait, wait, wait`

**Constructor Detail**

### NumericTransform

```
public NumericTransform()
```

:   Default constructor -- sets the default transform method
    to java.lang.Math.abs().


**Method Detail**

### globalInfo

```
public java.lang.String globalInfo()
```

:   Returns a string describing this filter

    :   **Returns:**: a description of the filter suitable for displaying in the explorer/experimenter gui

---


### getCapabilities

```
public Capabilities getCapabilities()
```

:   Returns the Capabilities of this filter.

    :   **Specified by:**: `getCapabilities` in interface `CapabilitiesHandler` **Overrides:**: `getCapabilities` in class `Filter`
    :   **Returns:**: the capabilities of this object **See Also:**: `Capabilities`

---


### setInputFormat

```
public boolean setInputFormat(Instances instanceInfo)
                       throws java.lang.Exception
```

:   Sets the format of the input instances.

    :   **Overrides:**: `setInputFormat` in class `Filter`
    :   **Parameters:**: `instanceInfo` - an Instances object containing the input instance structure (any instances contained in the object are ignored - only the structure is required). **Returns:**: true if the outputFormat may be collected immediately **Throws:**: `java.lang.Exception` - if the input format can't be set successfully

---


### input

```
public boolean input(Instance instance)
              throws java.lang.Exception
```

:   Input an instance for filtering. The instance is processed
    and made available for output immediately.

    :   **Overrides:**: `input` in class `Filter`
    :   **Parameters:**: `instance` - the input instance **Returns:**: true if the filtered instance may now be collected with output(). **Throws:**: `java.lang.IllegalStateException` - if no input format has been set.: `java.lang.reflect.InvocationTargetException` - if there is a problem applying the configured transform method.: `java.lang.NullPointerException` - if the input format has not been defined.: `java.lang.Exception` - if the input instance was not of the correct format or if there was a problem with the filtering.

---


### listOptions

```
public java.util.Enumeration listOptions()
```

:   Returns an enumeration describing the available options.

    :   **Specified by:**: `listOptions` in interface `OptionHandler`
    :   **Returns:**: an enumeration of all the available options.

---


### setOptions

```
public void setOptions(java.lang.String[] options)
                throws java.lang.Exception
```

:   Parses a given list of options.


    Valid options are:

    ```
     -R <index1,index2-index4,...>
      Specify list of columns to transform. First and last are
      valid indexes (default none). Non-numeric columns are 
      skipped.
    ```

    ```
     -V
      Invert matching sense.
    ```

    ```
     -C <string>
      Sets the class containing transformation method.
      (default java.lang.Math)
    ```

    ```
     -M <string>
      Sets the method. (default abs)
    ```

    :   **Specified by:**: `setOptions` in interface `OptionHandler`
    :   **Parameters:**: `options` - the list of options as an array of strings **Throws:**: `java.lang.Exception` - if an option is not supported

---


### getOptions

```
public java.lang.String[] getOptions()
```

:   Gets the current settings of the filter.

    :   **Specified by:**: `getOptions` in interface `OptionHandler`
    :   **Returns:**: an array of strings suitable for passing to setOptions

---


### classNameTipText

```
public java.lang.String classNameTipText()
```

:   Returns the tip text for this property

    :   **Returns:**: tip text for this property suitable for displaying in the explorer/experimenter gui

---


### getClassName

```
public java.lang.String getClassName()
```

:   Get the class containing the transformation method.

    :   **Returns:**: string describing the class

---


### setClassName

```
public void setClassName(java.lang.String name)
                  throws java.lang.ClassNotFoundException
```

:   Sets the class containing the transformation method.

    :   **Parameters:**: `name` - the name of the class **Throws:**: `java.lang.ClassNotFoundException` - if class can't be found

---


### methodNameTipText

```
public java.lang.String methodNameTipText()
```

:   Returns the tip text for this property

    :   **Returns:**: tip text for this property suitable for displaying in the explorer/experimenter gui

---


### getMethodName

```
public java.lang.String getMethodName()
```

:   Get the transformation method.

    :   **Returns:**: string describing the transformation method.

---


### setMethodName

```
public void setMethodName(java.lang.String name)
                   throws java.lang.NoSuchMethodException
```

:   Set the transformation method.

    :   **Parameters:**: `name` - the name of the method **Throws:**: `java.lang.NoSuchMethodException` - if method can't be found in class

---


### invertSelectionTipText

```
public java.lang.String invertSelectionTipText()
```

:   Returns the tip text for this property

    :   **Returns:**: tip text for this property suitable for displaying in the explorer/experimenter gui

---


### getInvertSelection

```
public boolean getInvertSelection()
```

:   Get whether the supplied columns are to be transformed or not

    :   **Returns:**: true if the supplied columns will be kept

---


### setInvertSelection

```
public void setInvertSelection(boolean invert)
```

:   Set whether selected columns should be transformed or not.

    :   **Parameters:**: `invert` - the new invert setting

---


### attributeIndicesTipText

```
public java.lang.String attributeIndicesTipText()
```

:   Returns the tip text for this property

    :   **Returns:**: tip text for this property suitable for displaying in the explorer/experimenter gui

---


### getAttributeIndices

```
public java.lang.String getAttributeIndices()
```

:   Get the current range selection

    :   **Returns:**: a string containing a comma separated list of ranges

---


### setAttributeIndices

```
public void setAttributeIndices(java.lang.String rangeList)
```

:   Set which attributes are to be transformed (or kept if invert is true).

    :   **Parameters:**: `rangeList` - a string representing the list of attributes. Since the string will typically come from a user, attributes are indexed from 1. eg: first-3,5,6-last **Throws:**: `InvalidArgumentException` - if an invalid range list is supplied

---


### setAttributeIndicesArray

```
public void setAttributeIndicesArray(int[] attributes)
```

:   Set which attributes are to be transformed (or kept if invert is true)

    :   **Parameters:**: `attributes` - an array containing indexes of attributes to select. Since the array will typically come from a program, attributes are indexed from 0. **Throws:**: `InvalidArgumentException` - if an invalid set of ranges is supplied

---


### getRevision

```
public java.lang.String getRevision()
```

:   Returns the revision string.

    :   **Specified by:**: `getRevision` in interface `RevisionHandler` **Overrides:**: `getRevision` in class `Filter`
    :   **Returns:**: the revision

---


### main

```
public static void main(java.lang.String[] argv)
```

:   Main method for testing this class.

    :   **Parameters:**: `argv` - should contain arguments to the filter: use -h for help


---


|  |  |  |
| --- | --- | --- |
| **Overview** \| **Package** \| **Class** \| **Tree** \| **Deprecated** \| **Index** \| **Help** \| **Weka's home** | |  |
| **PREV CLASS**   **NEXT CLASS** | **FRAMES**    **NO FRAMES**     **All Classes** |  |
| SUMMARY: NESTED \| FIELD \| CONSTR \| METHOD | DETAIL: FIELD \| CONSTR \| METHOD |  |


---
